# Identification of new MUC1 epitopes using HLA-transgenic animals: implication for immunomonitoring

**DOI:** 10.1186/s12967-017-1254-0

**Published:** 2017-07-05

**Authors:** Tanja Scheikl-Gatard, Caroline Tosch, François Lemonnier, Ronald Rooke

**Affiliations:** 1 SONOGEN AG, Badenerstrasse 808, 8048 Zurich, Switzerland; 2 Transgene SA, 400 Bld Gonthier d’Andernach, 67400 Illkirch Graffenstaden, France; 3 Unité INSERM 1016, Département Endocrinologie, Métabolisme et Diabète. Equipe Immunologie des Diabètes, Bâtiment Cassini, 123 Bd Port Royal, 75014 Paris, France; 4 Institut de Recherche Servier, 125 Chemin de Ronde, 78290 Croissy, France

**Keywords:** Cancer, Immunology, MHC class I, MUC1, Tumor antigen, Immunomonitoring

## Abstract

**Background:**

The success of immunotherapeutics in oncology and the search for further improvements has prompted revisiting the use of cancer vaccines. In this context, knowledge of the immunogenic epitopes and the monitoring of the immune response cancer vaccines generate are essential. MUC1 has been considered one of the most important tumor associated antigen for decades.

**Methods:**

To identify HLA-restricted MUC1 peptides we used eight human MHC class I transgenic mouse lines, together covering more than 80% of the human population. MUC1 peptides were identified by vaccinating each line with full length MUC1 coding sequences and using an IFNγ ELIspot restimulation assay. Relevant peptides were tested in a flow cytometry-based tetramer assay and for their capacity to serve as target in an in vivo killing assay.

**Results:**

Four previously identified MUC1 peptides were confirmed and five are described here for the first time. These nine peptide-MHC combinations were further characterized. Six gave above-background tetramer staining of splenocytes from immunized animals and three peptides were induced more than 5% specific in vivo killing.

**Conclusions:**

These data describe for the first time five new HLA class I-restricted peptides and revisit some that were previously described. They also emphasize the importance of using in vivo/ex vivo models to screen for immunogenic peptides and define the functions for individual peptide-HLA combinations.

**Electronic supplementary material:**

The online version of this article (doi:10.1186/s12967-017-1254-0) contains supplementary material, which is available to authorized users.

## Background

After many years of mitigated results, cancer immunotherapy approaches have spawned great enthusiasm because of their capacity to generate significant improvement in patients’ status in a number of pathologies [1]. One of these advances exploits immunological checkpoints for which commercially approved molecules prevent the dampening of the immune response arising in the tumor environment. These immune checkpoint inhibitors (ICI) have been successfully used as stand-alone in early clinical trials which indicates that effector T cells are present, that they are the main players in tumor control and that their incapacity to control cancer growth is due to tumor-related immunosuppressive mechanisms [2, 3]. It may also explain why cancer vaccines have met with limited success so far in that the tumor antigen-specific T cells they generate are incapable of fulfilling their task in face of an inhibitory tumor environment [4, 5]. Notwithstanding the significance of ICI in the treatment of cancer, only a proportion of patients respond. The mechanisms underlying the absence of response are multiple and many are currently being investigated [3]. One possible reason why some patients do not respond is the mere absence of an antigen-directed immune response by lack of stimulation of appropriate T cell clones. It is thereby reasonable to assume that the combination of cancer vaccines with ICI will increase the proportion of responding patients. Although important developments of sequencing technologies allow to foresee the use of patients idiotypic epitopes as source of antigens, the development path and regulatory hurdles of such technology jeopardizes their commercial success. Conversely, the use of a broadly distributed tumor antigen would justify the development of an “off the shelf” product as well as establishing the proof-of-concept that the immune responses stimulated by cancer vaccines are effective if the tumor-associated immune suppression is relaxed.

MUC1 is one of the most studied tumor associated antigen [6]. This mucin protein is highly distributed among cancers of epithelial origin and the cancer-associated post-translational modifications render it recognizable by the adaptive arm of the immune response. While it has been repeatedly identified as a major tumor-associated antigen, MUC1-targeting cancer vaccines have met with limited success in terms of patients’ benefit [7–11]. The immunomonitoring and biomarker identification programs that accompanied many studies have identified responder sub-populations in various cohorts [12, 13]. However, no consistent pattern of responders can be established. Moreover, while MUC1-specific immune responses have been seen in healthy donors, cancer patients and MUC1-vaccinated individuals by various means, to date, no correlation with the identified response and clinical outcome can be established [14, 15].

This may be accounted for by variations in the clinical protocols, in the choice of antigen and its delivery systems as well as differences in the monitoring methods. Because CD8+ T cells are believed to be the main effectors in tumor control and elimination, the identification of major histocompatibility complex class I (MHC I)-restricted peptides impacts on vaccine design and remains essential for monitoring purposes. Various in vitro and in silico methods have been developed to identify such peptides but their efficacy has been hampered by the heterogeneity of the human leukocyte antigen (HLA) distribution in the human population and the complexity of the antigen presentation machinery.

To identify MUC1 antigenic peptides, we made use of eight different HLA-transgenic mouse lines representing the most common human MHC class I alleles and covering approximately 80% of the human population [16]. We describe here for the first time, five HLA class I immunogenic peptides, each restricted to a specific HLA allele. Most of these peptides would not have been selected for immunomonitoring purposes by HLA-restricted peptide predicting algorithms. While these peptides were identified by their capacity to restimulate IFNγ production in vitro, only six out of nine corresponding peptide-HLA tetramers could detect CD8+ T cells after immunization. Further, when tested in an in vivo killing assay, only three peptides gave more than 5% specific cytotoxicity. The present work demonstrates that HLA transgenic animals are instrumental in identifying novel human epitopes that can then be used as source of antigen and/or for immunomonitoring. Each peptide performed differently in functional assays with no systematic qualitative or quantitative correlation across all assays, suggesting that they may be playing different roles in the immune response. These data warrant the use of HLA transgenic animals in combination with functional assays to better select immunogenic peptides for immunomonitoring or immunization approaches.

## Methods

### Mice

The monochain homozygous HLA-transgenic mice have been described previously [16–19]. Four digit alleles used to create each line are listed in Table 1. Mice were kept under specific pathogen-free conditions with water and food ad libitum. This study was conducted in compliance with European Union (EU) directive 2010/63/EU for animal experiments. An institutional ethical committee has approved the experiments performed in this study.

**Table 1:** HLA transgenic mouse strains used and associated MUC1 peptides

Mouse HLA	Identified peptide	AA position in the MUC1 protein^a^	Identification method	References
HLA-A*01	is **E** mflqi **Y**	1123–1131	Binding assay	[13]
HLA-A*02	stappvhn **V**	950–958	Prediction program	[22]
	s **L** sytnpa **V**	1240–1248	Binding assay	[13]
	l **L** ltvlt **V**	14–21	Prediction program	[23]
	v **L** vcvlva **L**	1165–1173	New	
HLA-B*07	a **P** dnrpa **L**	941–948	New	
HLA-B*27	r **R** knygqldi **F**	1187–1197	New	
HLA-B*35	f **P** ardtyhp **M**	1197–1206	New	
HLA-C*07	difp **A** rdt **Y**	1195–1203	New	

Anchor residues are in bold

^a^Peptide numbering is based on the Uniprot sequence P15941-1 refered to as the canonical sequence

### MUC1 peptide pool library

Peptides were synthesized by ProImmune (Oxford, UK) or ProteoGenix (Schiltigheim, France) to a minimum purity of 95%. The identity of each peptide was confirmed by mass spectral analysis. The peptide libraries cover the entire MUC1 protein sequence and are either composed of 11mers overlapping by 8 amino acids or 15mers overlapping by 11 amino acids. All peptides were suspended in DMSO at a concentration of 50 µg/mL. To identify HLA-specific antigenic peptide, peptides of the same length were pooled to a final concentration of 50 µg/mL per peptide (25 pools of 12 or 13 peptides for the 11mers and 24 pools of 11 or 12 peptides for 15mers). Pools were used in a matrix format as described in Tobery [20].

### MUC1-immunizing vectors

MUC1 plasmid was generated by introducing a modified sequence of the human MUC1 cDNA (NCBI Nucleotide database# NM_002456.5) into the pcDNA3.1ΔHygro expression vector. The plasmid preparation and purification was done by Geneart (ThermoFisher, Courtaboeuf, France). The plasmid was stored at 4 °C in TE buffer (10 mM TRIS, 0.1% EDTA, pH 8) and diluted in PBS prior to use. A Modified Vaccinia Ankara (MVA) recombinant virus expressing the MUC1 protein (NCBI Nucleotide database# NM_002456.5) was generated by homologous recombination between the two expression cassettes and the empty vector MVATGN33.1 in primary chicken embryo fibroblasts (CEFs) as described earlier [21]. The purified virus was suspended in S08 buffer (10 mM Tris–HCl, pH 8, 5% (wt/vol) sucrose, 10 mM sodium glutamate, and 50 mM NaCl) and stored at 80 °C. Virus stocks between 5 × 10^8^ and 10^9^ PFU/mL as determined by CEF-plaque assay. Viruses were diluted in S08 buffer to the concentrations required for the in vivo studies immediately prior to use.

### Immunizations

For DNA immunization, anesthetized mice were first injected intramuscularly in both tibialis anterior muscles with cardiotoxin (50 µL at 10 µM, Latoxan, Rosans, France) then, 5–7 and 17–21 days later, they were injected at the same site with 50 μg per leg of purified recombinant MUC1 plasmid DNA in 50 µL. For MVA immunization, mice were injected intravenously with 5 × 10^7^ PFU of recombinant MVA-MUC1 in a final volume of 100 μL. MVA was injected twice or thrice with a 7-day interval between injections. Epitope-specific CD8+ T cell responses were analyzed 7–9 days after the last injection of either MVA or DNA.

### ELISpot assay

CD4-depleted (mouse CD4 MicroBeads, Miltenyi Biotec, Paris, France) or CD8-enriched (CD8α T cell isolation kit II; Miltenyi Biotec, Paris, France) splenocytes (2–5 × 10^5^ cells) were seeded in triplicate wells in 96-well polyvinylidene difluoride (PVDF) membranes (MultiScreen HTS; Millipore, Fontenay Sous Bois, France) previously coated with a rat anti-mouse anti-IFNγ mAb (15 μg/mL, AN-18; Mabtech, Paris, France) and cultured in RPMI-1640 supplemented with 10% Fetal Calf serum in the presence of 1 µg/mL of MUC1 peptide pools or individual peptides. After 18 h culture, IFNγ-producing cells were revealed using biotinylated rat anti-mouse anti-IFNγ detection mAb (1 μg/mL, R4-6A2-biotin; Mabtech, Paris, France), ExtrAvidin-alkaline phosphatase (1:5.000; Sigma-Aldrich, Paris, France) and BCIP/NBT solution (Sigma-Aldrich, Paris-France). Spots were counted using Bioreader 4000 PRO-S and analyzed with the ImmunoSpot software (BIO-SYS, Karben, Germany). Background values were defined as mean number of spots obtained in absence of antigenic peptides + 2× the standard deviation and subtracted from the values obtained with antigenic peptides.

### Flow cytometry

1–2 × 10^6^ total splenocytes or CD8-enriched T cells (CD8α T-cell isolation kit II; Miltenyi Biotec, Paris, France) were incubated for 30 min at room temperature with 2.5 μL of phycoerythrin (PE)-conjugated HLA-MUC1-peptide tetramer (TC Metrix, Epalinges, Switzerland) or chimeric (HLA-A2 α1 + 2, H2Kb α3 or HLA-B7 α1 + 2, H2Db α3) MUC1-peptide containing tetramer (S. Buus, Copenhagen University, Denmark). Cells were then stained with NearIR LIVE/DEAD marker (Molecular Probes, Paris, France) for 15 min. After washing, cells were incubated for 30 min at 4 °C with anti-CD8 (RM4-5), anti-CD4 (53–6.7), anti-NK1.1 (PK136), anti-B220 (30-F11) and anti-CD11b (M1/70) antibodies (all BD Biosciences, Pont-de-Claix, France). Except for the anti-CD8 Ab which was APC-conjugated, all Ab were FITC conjugated and used as dump-channel.

Data was acquired using a FACS Aria III (BD Biosciences) and analyzed using FlowJo 7.6.1 software (Tree Star, Ashland, OR, USA) or Kaluza (Beckman Coulter, Villepinte, France).

### In vivo cytotoxicity assay

For in vivo CTL activity, splenocytes from naive HLA-matched animals were divided into several groups; unpulsed or pulsed for 1 h at 37 °C with 10 μM of relevant MUC1 peptides and labeled with 0.4 or 6.4 μM carboxyfluorescein succinimidyl ester (CFSE) (Invitrogen, Paris, France) for 10 min at 37 °C and/or 30 min at 37 °C with 1 µM CellTracker™ Orange CMTMR (6-(((4-chloromethyl)benzoyl)amino)tetramethylrhodamine) (1:1000, Molecular Probes, Paris, France). After washing, the different fractions were mixed in equal proportions for intravenous injection into recipient mice with a maximum of 3 × 10^7^ cells per mouse. Splenocytes were harvested 18 h later, prior to flow cytometry acquisition. The percentage of specific lysis was calculated using the formula: % specific lysis = 100 – [100 × (R in immunized mice/mean R in naive mice)], where R is the ratio of the number of pulsed cells/number of unpulsed cells.

Data were acquired either on a FACSCanto, a FACSAria III flow cytometer (Becton, Dickinson) or a Navios cytometer (Beckman Coulter). Accordingly, analyses were performed with Diva or Kaluza (Beckman Coulter) software.

### Statistical analyses

Mann–Whitney tests were performed for individual comparisons of two independent groups. Wilcoxon tests were performed for individual comparisons of paired groups. Statistical analysis was performed with GraphPad Prism (version 5) software. Differences were considered significant at P values of <0.05.

## Results

### Identification of novel HLA-restricted MUC1 specific peptides

**Fig. 1:**
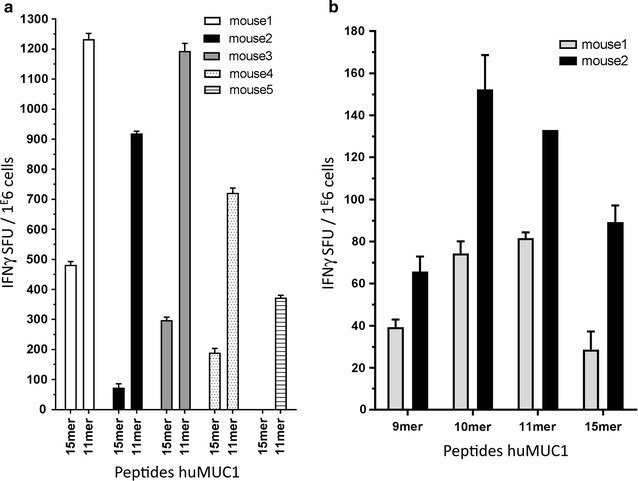
Representative results for IFNγ Elispots showing CD8-specific MUC1 responses. **a** Splenocytes from five immunized HLA-B*27 mice restimulated with 11mer R**R**KNYGQLDI**F** gave a stronger response than the 15mer R**R**KNYGQLDI**F**PARD (anchor residues in *bold*). **b** CD4-depleted splenocytes from two different immunized HLA-B*27 mice were restimulated with 9mer **R**KNYGQLDI, 10mer R**R**KNYGQLDI, 11mer R**R**KNYGQLDI**F** and the identified 15mer R**R**KNYGQLDI**F**PARD from the peptide pool. The 11mer was chosen for the tetramer construction as it gave a strong INFγ response and contains both anchor residues for HLA-B*27 (marked in *bold*)

To identify MHC class I-restricted peptides, mice from each HLA-transgenic line were immunized by intramuscular injection of a plasmid encoding the entire MUC1 sequence. Following the immunization protocol, the frequency of IFNγ-producing splenocytes was determined after stimulation with pools of 15mer peptides overlapping by 11 amino acids. The peptide pools used for the stimulation were composed and displayed in a matrix format allowing identification of individual peptides (Additional file 1: Figure S1) [20]. Six out of the eight mouse lines immunized with the MUC1-expressing plasmid gave an “above background” number of IFNγ-producing cells suggesting that some of the 15mer comprise the human MHC-restricted peptide (not shown). As described by Boucherma et al. all mouse lines have a broad T cell receptor (TCR) repertoire representative of a normal CD8 compartment exclusively selected and maintained by the transgenic human MHC class I molecule since the H2 MHC class I locus is inactivated [16]. Notwithstanding this observation, the proportion of CD8+ T cells present in the lymphoid compartment in the HLA-transgenic lines is lower than the one seen in inbred but otherwise wild type animals. To enhance the proportion of peptide pool-stimulated cells, we either enriched for the CD8+ cells or depleted the CD4+ cells prior to the ELISpot assay. Although this generated more interpretable results, the use of 15mers was often associated with inconsistencies in the matrix analysis (Additional file 1: Figure S1a). Fifteen-mer peptides must be randomly trimmed by proteases present in the medium to fit in the groove of MHC class I molecules or can be presented by MHC class II molecules, thereby stimulating CD4+ T cell. We reasoned that to better discriminate the MUC1 peptides capable of stimulating a MHC class I response, splenocytes from immunized animals were screened with the pools of 11mer peptides, overlapping by eight amino acids and covering the entire MUC1 sequence (Fig. 1a). The combination of cell enrichment and use of 11mers increased the proportion of responding cells per seeded cells and limited the detection of mouse class II-restricted response respectively (Additional file 2: Figure S2).

In any case, for every 15 or 11mer peptide generating an above-background response, a restimulation assay was done with peptides of various lengths (8–11mers) spanning the suspected MUC1 region considering the HLA-specific anchor residues (Fig. 1b). Table 1 summarize the peptides that generated the strongest responses per transgenic mouse line. We confirmed in the HLA-A*01 mouse line a peptide originally identified using a binding assay [13]. Genetic immunization of HLA-A*02 mice also confirmed three peptides previously described identified either using a binding assay (SLSYTNPAV) [13] or Peptide Prediction Algorithms (STAPPVHNV, LLLTVLTVV) [22, 23]. Interestingly, a hereto forth undescribed HLA-A*02-restricted peptide was identified with our approach (VLVCVLVAL). Since we focused on the peptides that generated the strongest ELISpot responses, many other HLA-A*02-restricted peptides described in the literature were not further analyzed as they only generated marginal responses. Finally, we identified one novel HLA class I-restricted peptide for each of the additional mouse lines listed in table I. No MUC1-specific response could be generated in the HLA-A*24 and HLA-B*08 mouse lines (data not shown) [16].

### HLA-specific tetramers staining and limitations of peptide-HLA predicting algorithms

**Fig. 2:**
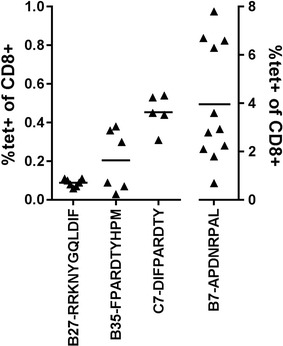
Tetramer staining. Splenocytes of immunized mice were stained with the respective HLA-tetramer. Each *triangle* represents the percentage of tetramer positive CD8+ cells per mouse. *Horizontal bars* represents the mean of all animals. A1-ISEMFLQIY, A2-STAPPVHNV and A2-VLVCVLVAL gave no tetramer staining (not shown)

**Fig. 3:**
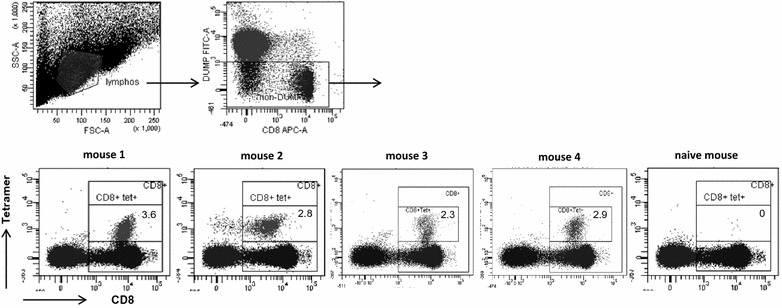
Representative tetramer staining of HLA-B*07 splenocytes. Splenocytes of immunized HLA-B*07 mice (mouse 1–4) or 1 naive mouse were analysed for B7-APDNRPAL tetramer staining. Lymphocytes were gated as described in “Methods”. *Numbers* represent proportion of tetramer+ in the total CD8+ population

Fluorescence-conjugated peptide-HLA tetramers are important tools for monitoring the evolution of an antigen-specific immune response. To determine if direct detection of the peptide-MHC responding CD8+ cells was possible, tetramers were produced for each combination and tested on splenocytes of immunized animals (Fig. 2). Globally, of the nine tetramers made with fully human MHC molecules tested, only three did not give any staining. Of those, the HLA-A*01-restricted peptide (ISEMFLQIY) which was originally identified using a HLA binding assay [13] was predicted as the best binder by several peptide-prediction algorithms (not shown) and it systematically gave a high frequency of IFNγ-producing cells in our restimulation assay. Two out of the four HLA-A*02 tetramers did not detect CD8+ cells from immunized animals although each peptide generated a significant IFNγ response in the ELIspot assay. One of these peptides (STAPPVHNV) has been previously shown to induce a cytotoxic response in a number of studies [15, 22, 24]. To our knowledge, only one study used STAPPVHNV-HLA-A*02 tetramers to look at MUC1-specific CD8+ cells in the blood of healthy individuals and cancer patients and found very low frequencies [25]. Interestingly, four out of five different peptide-HLA binding prediction algorithms predicted low binding capacity for this peptide (Additional file 3: Table S1). On the other hand, the same algorithms predict a higher probability for the novel VLVCVLVAL peptide to bind HLA-A*02 although we were incapable of detecting any significant tetramer staining. In the HLA-B*07 mouse line, the peptide identified (APDNRPAL) has never been described before. This is not surprising since only 2 out of 5 peptide-binding prediction algorithms could analyze the binding capacity of 8mers and did not rank it as the one showing the highest affinity. This is also in sharp contrast with the high intensity and high frequency of staining obtained with this tetramer on CD8+ splenocytes from immunized animals (Figs. 2, 3). Similarly, only two programs predicted the binding capacity of 11 mers. However, they both ranked the novel HLA-B*27 binding peptide (RRKNYGQLDIF) as the peptide with the highest affinity which correlated with the tetramer staining. Finally, the HLA-B*35 and HLA-C*07 restricted peptides (FPARDTYHPM and DIFPARDTY, respectively) are both described for the first time. The HLA-B*35-binding peptide was identified by all algorithms in the top six high affinity peptides, ranging from 1st to 6th and correlated with tetramer staining. In contrast, the HLA-C*07-restricted peptide was described as a low affinity by the algorithms but nonetheless showed good tetramer staining. In conclusion, the use of HLA-expressing transgenic animals allowed the identification of novel HLA-binding peptides which would not have been identified using binding algorithms. Even if some predicted peptides map to the repeated sequences in the MUC1 sequence, all the peptides identified as immunodominant in the assay are unique in the protein sequence (Table 1).

### Tetramer avidity impacts on the detection of CD8+ cells

**Fig. 4:**
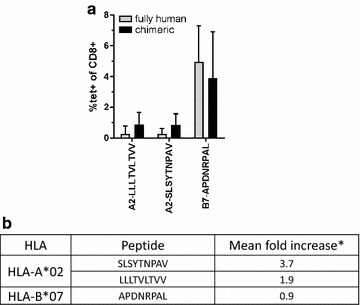
Fully human vs chimeric tetramer staining. Fully human HLA molecules or human-mouse chimera (human α1 + α2 + mouse Db α3) were loaded with the indicated peptide and used to stain splenocytes from immunized animals. **a** Mean percentage of stained CD8+ cells (n = 5 mice for fully human tetramer, n = 4 for chimeric constructs). **b** Fold increase in percentage of chimeric tetramer stained CD8+ cells/percentage of fully human tetramer stained CD8+ cells

From the data described above, it appears that some peptide-MHC complexes stimulate the production of IFNγ but that the tetramers made of the same constituents are incapable of staining specific T cells. Many parameters impact on the interaction between clonotypic TCRs and their cognate peptide-MHC heterodimer which have led to the concept of functional avidity (see [26] for review). An important component influencing the TCR-peptide-MHC (pMHC) avidity, both in vitro and in vivo, is the CD8 heterodimeric co-receptor binding to the α3 domain of MHC class I molecules [26–30]. One possible explanation for the discrepancy between the positive ELIspot data and the absence of tetramer staining observed for some peptides may be related to the fact that the CD8+ T cells in HLA-transgenic mouse lines have been selected on chimeric MHC class I molecules harboring a mouse D^b^ α3 portion while the MHC class I molecules making up the tetramer are fully human. The contribution of the CD8-MHC class I interaction has been estimated to affect the TCR avidity by a factor of three to fourfold [28]. To address this possibility, tetramers made of chimeric HLA-A*02 and HLA-B*07 molecules corresponding precisely to the ones expressed in the animals were synthetized, loaded with the appropriate peptides and used to stain splenocytes from immunized animals. Figure 4 shows the percentages of stained CD8+ cells of immunized animals with the corresponding tetramers that could be successfully assembled. The proportion of CD8+ splenocytes stained by the fully human or chimeric HLA-B*07-APDNRPAL tetramers were not significantly different which argues in favor of a strong affinity between the TCR and this pMHC. On the other hand, while the four fully human HLA-A*02 tetramer were synthesizable, only two out of four chimeric HLA-A*02 tetramers could be obtained. The chimeric HLA-A*02 tetramers gave systematically a higher percentage of stained cells and in some mice, they allowed measuring a response otherwise undetectable with the fully-human tetramer. These latter observations suggest that the increase in avidity generated from the α3-CD8 interaction is important in the detection of a HLA-A*02 responses.

### Novel peptides as target for a cytotoxic response

**Fig. 5:**
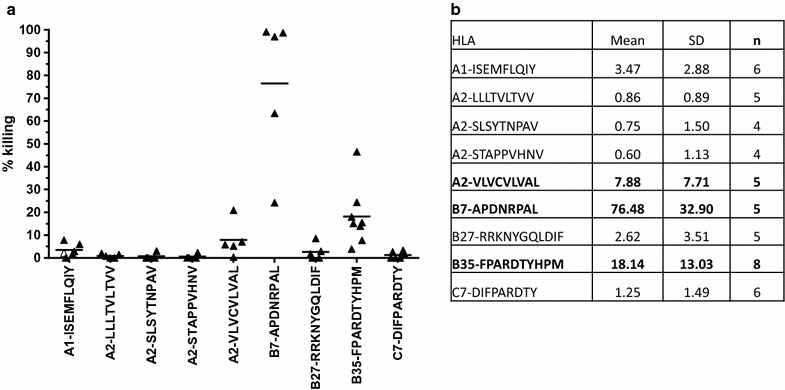
In vivo cytotoxicity. Fluorescence-labelled splenocytes of HLA-matched mouse line were loaded with the indicated peptide and injected in either immunized or naive mice. Animals received concomitantly untreated splenocytes (no peptide) labelled with a different concentration of the fluorescent label. Killing was determined as described in “Methods”. **a** Each *triangle* represents the percentage of specific killing per mouse. *Horizontal bar* mean of all mice. **b** Table summarizing mean percentage of cytotoxicity and standard deviation. *n* number of animals analyzed. Peptides B7-ADPNRPAL, B35-FPARDTYHPM and A2-VLVCVLVAL showed a mean specific killing above 5% (shown in *bold*)

We then evaluated the cytotoxic capacity of the CD8+ cells generated in immunized animals towards peptide-loaded target cells in an in vivo cytotoxic assay. Since background killing, defined as the difference between the percentages of killing in untreated splenocytes in immunized and naïve animal, was negligible in all experiments (no non-specific killing), we considered 5% cytotoxicity as the threshold for positivity. From this standpoint, three newly identified MUC1 peptides (HLA-A*02-VLVCVLVAL, HLA-B*07-ADPNRPAL and HLA-B*27-RRKNYGQLDIF) were capable of inducing cell killing in vivo (Fig. 5). For each mouse line, variation in cytotoxicity was important between animals (5 < N < 8, N = number of mice evaluated) but most values remaining within the same quartile. However, cytotoxicity varied greatly between mouse lines with mean values ranging from <1% (HLA-A*02-LLLTVLTVV) to >75% (HLA-B*07-ADPNRPAL). In the latter case, some animals eliminated the peptide loaded target and demonstrated 100% cytotoxicity. Many potentially additive mechanisms can be responsible for these differences such as peptide half-life on the surface of loaded cells, the functionality of the amplified CD8+ T cell clone and/or the in vivo effector to target ratio. It is important to note that we established positivity on the mean cytotoxicity value obtained from many animals (5 < N<8) which includes animals which may not have been successfully immunized. The data presented may thus be underestimated.

## Discussion

Cancer immunotherapy has been the subject of research and speculations for over a century and it is only in the first half of this decade that clinical data has demonstrated its efficacy. Numerous ways to use patient’s immune system against his/her cancer have been put forth and tested (reviewed in [31]). To date, the most successful approach is based on antibodies that block signals naturally used by the immune system to control the breadth of the immune response and prevent autoimmunity. Four such ICI antibodies, targeting two main, complementary inhibitory pathways are approved by the FDA in various indications: ipilimumab blocks the interaction between the cytotoxic T lymphocyte associated protein 4 (CTLA-4) on T cells and the CD80/CD86 molecules on the antigen presenting cells (APC) while nivolumab, pembrolizumab and atesolizumab are inhibitors of the interaction between the programmed death 1 (PD-1) receptor and its ligand (PD-L1). The other FDA-approved approach in cancer immunotherapy consists in vaccinating patients against their cancer [32]. It is based on the delivery of peptides or proteins that are specific to the cancer cells in a context that stimulates an immune response against the antigen-expressing cells [4]. Conceivably, the combination of ICI with vaccination approaches are complementary and should result in improved responses in the ongoing clinical trials [33]. This concept is exemplified by data stemming from clinical trials which show that patients responding to treatment with ICI have high mutation rates. This is interpreted as the demonstration that patients with tumors displaying a broad range of neo-epitopes are more likely to develop effector T cell responses since they have not been subject to central tolerance. Moreover, these responses tend to be more efficient when the neoepitope-encoding mutations are homogeneously distributed across the tumor [34]. In this context, improved cancer vaccines may be designed and used to favor therapeutic benefit [4].

Evaluating the immune response of cancer patients has been highly instrumental for our understanding of the interplay between the immune system and patients’ cancers. The paradigm being established changes our view in the staging of patients and impacts on treatment choice [35]. It also places CD8+ effector T cells as a central component of an effective anti-cancer response further emphasizing the role therapeutic vaccination could play in conditions of relieved immunosuppression. In clinical trials, many therapeutic vaccination schemes were shown to generate specific responses to the antigen but correlation with an objective tumor control was seldom reported [36]. Great efforts were invested to identify the relevant response and ensure the robustness and comparability of the method to evaluate it through international proficiency panels [37, 38]. Flow cytometry offers the possibility of timely quantitative and qualitative assessments of immune responses throughout the course of diseases and/or treatments. The use of fluorescent pMHC multimers in combination with antibody cocktails that detect surface markers and secreted molecules allows enumerating antigen-specific T cells with specific phenotypes. However, the knowledge of the patients’ MHC haplotype, the determination of the peptide presented by specific HLA molecules and the capacity to synthesize and validate the pMHC multimer are paramount to this endeavor. The main constraint in achieving this goal has been the limited capacity of laboratory and bio-informatics tools to recapitulate all the steps involved in antigen presentation and thereby predicting and/or identifying antigenic peptides recognized by specific T cells. Here we show that an ensemble of eight mouse strains, each expressing a single HLA molecule together present in more than 80% of the human population, is crucial for the proper identification of antigenic peptides. In addition, it allows the evaluation/validation of the tools required to measure the antigen-specific response. We used the MUC1 protein as a model TAA since it is one of the most commonly expressed protein on tumor of epithelial origin and is the target of many immunotherapeutic vaccination protocols. In addition to its increased expression level and the loss of its apical expression pattern on tumor cells, MUC1 is recognized as a tumor-associated antigen following post-translational modifications rather than mutations in its coding sequence [7]. This characteristic makes it a public antigen more compatible with an “off-the shelf” therapeutic vaccine development scheme. The changes in the glycan moieties contribute to the enhanced immunogenicity of MUC1 by exposing the core protein to the humoral response and by modifying the interaction of cancer cells with APC. It is interesting to note that all the peptides we have identified lie outside of tandem repeat sequence that comprises all the O-linked glycosylation sites.

The method presented here identified five new antigenic peptides that have not been identified by antigenic peptide predicting algorithms nor by in vitro methods. Moreover, it allows rapid and unequivocal identification of peptides that best fit the MHC groove with limited steps to define the proper length. This is best exemplified by the identification of a novel HLA-A*02 peptide (VLVCVLVAL) despite the extensive work and the spectrum of tools used to identify HLA-A*02-restricted peptides. The demonstration that the most immunogenic peptide was a HLA-B*07-restricted 8mer (APDNRPAL) unpredictable by three out of five bio-informatic tools is another illustration of the usefulness of this method.

The use of HLA-transgenic animals also offers the possibility of evaluating the performance of pMHC-tetramers prior to their use in humans. Immunomonitoring of patients accrued in clinical trials represents important additional logistics and associated costs. This justifies upstream validation of the tools and methods. HLA-transgenic animals may be an important asset to achieve this. Indeed, our results show that of the nine peptides identified for their capacity to induce IFNγ production in a restimulation assay, only six pMHC-tetramers could detect Ag-specific CD8+ cells. The HLA molecule expressed in the animals are chimeras made of the α1 and α2 domains from the human sequence fused the α3 domain of the mouse H2 D^b^ molecule. This construct was shown to impact favorably on positive selection and maintenance of CD8+ cells in HLA-expressing animals by permitting a better interaction between the transgenic protein and the mouse CD8 molecule with negligible impact on the structure of the peptide-binding pocket [39, 40]. The loss of this interaction impacts on the avidity of the TCR-pMHC interaction and may be a possible explanation as to why cells induced to produce IFNγ in an ex vivo assay remained undetectable when exposed to a MHC tetramer containing the stimulatory peptide. To address this point, we loaded the same peptide in either tetramers made of fully human or chimeric HLA molecules and compared their capacity to detect the splenocytes coming from the same animals. Although results did not reach significance, for each combination studied, the chimeric tetramer detected a higher percentage of CD8 cells than the tetramers made of fully human HLA. These results are in line with the ones published by Choi et al. justifying the use of chimeric tetramers when using HLA transgenic animals to monitor the Ag-specific immune response [41].

HLA-transgenic mice offer the possibility to further characterize the immune response generated by immunogenic peptides by performing functional assays. Here, we have examined the cytotoxic response in immunized animals independent of our capacity to detect a tetramer-specific population. The results summarized in Table 2 show that it was not possible to establish a correlation between tetramer staining and in vivo cytotoxicity.

**Table 2:** Compilation of peptide identification and validations methods

Mouse HLA	Peptide	Tetramer		In vivo kill
		Fully human	Chimeric	
HLA-A*01	is **E** mflqi **Y**	Neg	ND	Neg
HLA-A*02	stappvhn **V**	Neg	ND	Neg
	s **L** sytnpa **V**	4/6	4/4	Neg
	l **L** ltvlt **V**	1/6	4/4	Neg
	v **L** vcvlva **L**	Neg	ND	4/5
HLA-B*07	a **P** dnrpa **L**	11/11	5/5	5/5
HLA-B*27	r **R** knygqldi **F**	Neg	ND	Neg
HLA-B*35	f **P** ardtyhp **M**	Neg	ND	8/8
HLA-C*7	difp **A** rdt **Y**	Neg	ND	Neg

All pMHC gave above background IFNg-Elispot in an in vitro recall response in at least three independent experiments. For tetramer staining and in vivo kill assay, the number of positive results (number of mice positive/total number of mice analyzed). Anchor residues are in bold

*Neg* negative results, *ND* not done

Of the nine peptides identified for their capacity to stimulate splenocytes of immunized mice in an IFNγ ELIspot assay, six could specifically stain CD8+ T cells in a tetramer assay and three were recognized as target by cytotoxic T cells in vivo. Conversely, one of these peptides induces killing even though the tetramer was unsuccessful at recognizing a specific T cell population. Some technical constraints may explain, at least in part these results, namely the fact that some mouse strains have only a partially reconstituted CD8+ compartment and that the ratio of target to effector cell may be important in the in vivo killing assay even though no trend was seen.

In terms of product development, the use of a tumor-specific antigen common to multiple tumor types and demonstrating little polymorphism between individuals offers advantages over personalized vaccination schemes that are currently in development.

## Conclusions

The results presented here describe novel MUC1 peptides that should be included in the immunomonitoring of patients. We also demonstrate the superiority of HLA-transgenic mouse lines over in vitro or in silico methods to identify novel peptides of well-studied tumor associated antigen. One important advantage is the possibility of performing functional assays. The fact that peptide-MHC complexes generate a range of response by clonotypic T cells emphasizes the importance of performing multiple assay to better define the role played by immunogenic peptides and these points are best addressed using HLA-transgenic animals.

## Additional files

**Additional file 1: Figure S1.** Representative IFNγ Elispot restimulation results and peptide pool matrix analysis. HLA-C*07 mice were immunized with full length MUC1 coding sequences and assay was performed on splenocytes. (a) 15mer. CD8-enriched cells (over 44% CD8+ cells) pooled from four immunized mice (HLA-C*07) were restimulated with pool of 15mers 1–24. The Elsipot is shown above, the matrix of 15mers 1–137 from the 24 pools covering the whole MUC1 protein is shown below. Pools above background in the Elispot (framed) are highlighted in the matrix. (b) 11mer. CD8-enriched cells (over 65% CD8+ cells) pooled from 7 immunized mice (HLA-C*07) were restimulated with pool of 11mers 1–25. The Elispot is shown above, the matrix of 11mers 1–156 from the 25 pools covering the whole MUC1 protein is shown below. Pools above background in the Elispot (framed) are highlighted in the matrix.

**Additional file 2: Figure S2.** CD8-specific IFNγ response. Total splenocytes (grey with 23% of CD8+ and 14.4% CD4+), CD8-enriched cells (black with 84% CD8+, 0.2% CD4+) and CD8-depleted cells (white with 1.6% CD8+ and 19.6% CD4+) pooled from four immunized mice (HLA-B*35) were restimulated with 10mer FPARDTYHPM and 11mer IFPARDTYHPM. Only cells containing CD8+ cell showed an IFNg response (grey and black), the CD8-depleted cells (white, not visible) showed no IFNγ response.

**Additional file 3: Table S1.** Prediction of antigenic peptide by algorithms. The referenced MUC1 sequence was submitted to five algorithms for antigenic peptide prediction. Default settings were used for each of them. Numbers represent the rank for each indicated peptide. “-” indicates that the algorithm could not predict the peptide. Colored box indicate that the algorithm lacked one or more conditions for analysis (peptide length and/or allele).

## Abbreviations

APC: allophycocyanine
CEF: chicken embryo fibroblasts
CFSE: carboxyfluorescein succinimidyl ester
CMTMR: 6-(((4-chloromethyl)benzoyl)amino)tetramethylrhodamine
CTL: cytotoxic T lymphocyte
FITC: fluoresceine iso-thiocyanate
HLA: human leukocyte antigen
ICI: immune checkpoint inhibitors
IFNγ: interferon gamma
mAb: monoclonal antibody
MHC: major histocompatibility complex
MUC1: mucin 1
MVA: Modified Vaccinia Ankara Strain
PBS: phosphate buffered saline
PE: phycoerythrine
