# Supplementary figures and images for: Identification of new MUC1 epitopes using HLA-transgenic animals: implication for immunomonitoring
Source: J Transl Med. 2017 Jul 5;15:154. doi: 10.1186/s12967-017-1254-0 (PMC5499006; doi:10.1186/s12967-017-1254-0)

## Slide 1
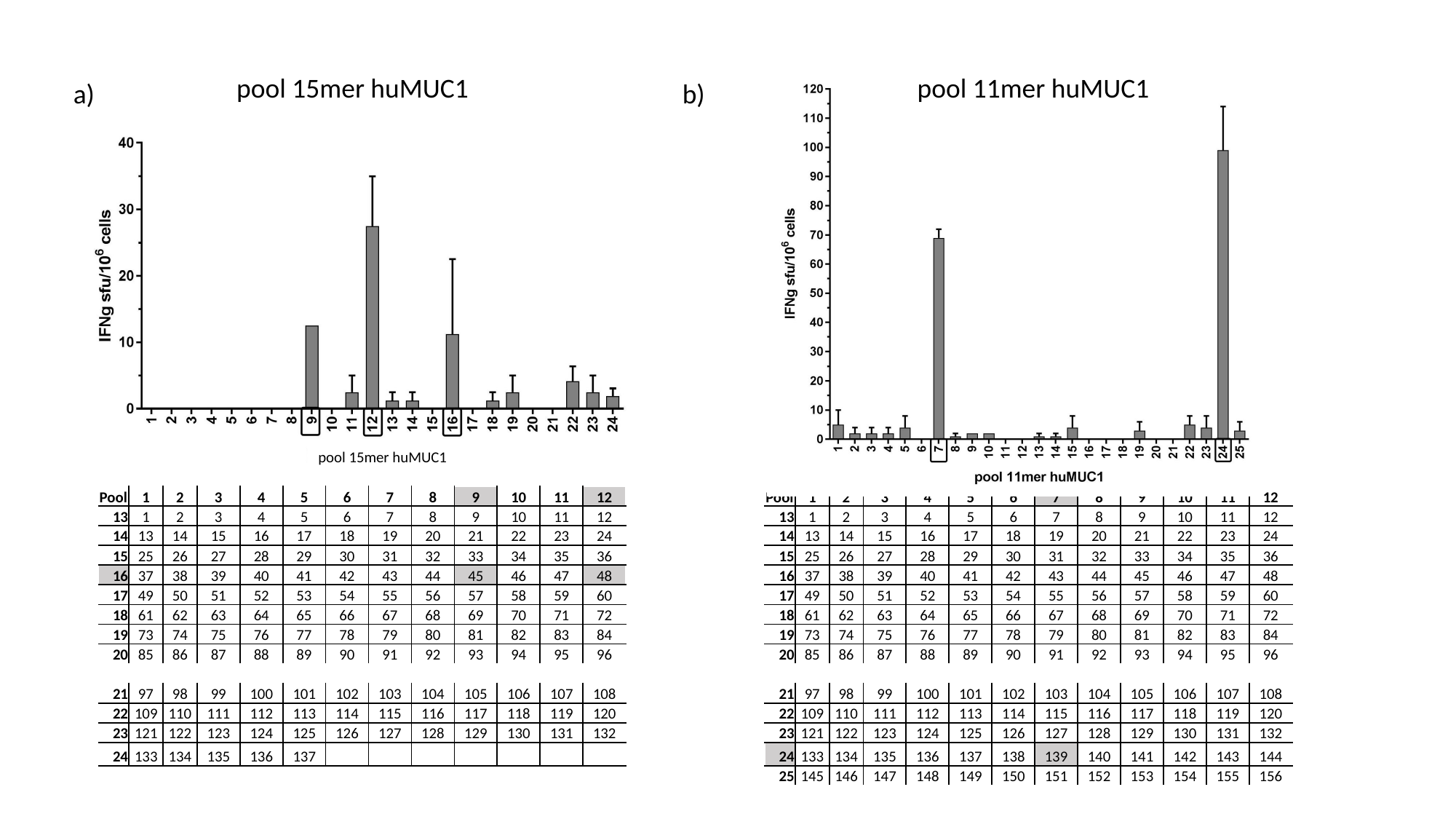

pool 15mer huMUC1
pool 11mer huMUC1
a)
b)
pool 15mer huMUC1
| | | | | | | | | | | | | | |
| --- | --- | --- | --- | --- | --- | --- | --- | --- | --- | --- | --- | --- | --- |
| | Pool | 1 | 2 | 3 | 4 | 5 | 6 | 7 | 8 | 9 | 10 | 11 | 12 |
| | 13 | 1 | 2 | 3 | 4 | 5 | 6 | 7 | 8 | 9 | 10 | 11 | 12 |
| | 14 | 13 | 14 | 15 | 16 | 17 | 18 | 19 | 20 | 21 | 22 | 23 | 24 |
| | 15 | 25 | 26 | 27 | 28 | 29 | 30 | 31 | 32 | 33 | 34 | 35 | 36 |
| | 16 | 37 | 38 | 39 | 40 | 41 | 42 | 43 | 44 | 45 | 46 | 47 | 48 |
| | 17 | 49 | 50 | 51 | 52 | 53 | 54 | 55 | 56 | 57 | 58 | 59 | 60 |
| | 18 | 61 | 62 | 63 | 64 | 65 | 66 | 67 | 68 | 69 | 70 | 71 | 72 |
| | 19 | 73 | 74 | 75 | 76 | 77 | 78 | 79 | 80 | 81 | 82 | 83 | 84 |
| | 20 | 85 | 86 | 87 | 88 | 89 | 90 | 91 | 92 | 93 | 94 | 95 | 96 |
| | | | | | | | | | | | | | |
| | 21 | 97 | 98 | 99 | 100 | 101 | 102 | 103 | 104 | 105 | 106 | 107 | 108 |
| | 22 | 109 | 110 | 111 | 112 | 113 | 114 | 115 | 116 | 117 | 118 | 119 | 120 |
| | 23 | 121 | 122 | 123 | 124 | 125 | 126 | 127 | 128 | 129 | 130 | 131 | 132 |
| | 24 | 133 | 134 | 135 | 136 | 137 | | | | | | | |
| | | | | | | | | | | | | | |
| --- | --- | --- | --- | --- | --- | --- | --- | --- | --- | --- | --- | --- | --- |
| | Pool | 1 | 2 | 3 | 4 | 5 | 6 | 7 | 8 | 9 | 10 | 11 | 12 |
| | 13 | 1 | 2 | 3 | 4 | 5 | 6 | 7 | 8 | 9 | 10 | 11 | 12 |
| | 14 | 13 | 14 | 15 | 16 | 17 | 18 | 19 | 20 | 21 | 22 | 23 | 24 |
| | 15 | 25 | 26 | 27 | 28 | 29 | 30 | 31 | 32 | 33 | 34 | 35 | 36 |
| | 16 | 37 | 38 | 39 | 40 | 41 | 42 | 43 | 44 | 45 | 46 | 47 | 48 |
| | 17 | 49 | 50 | 51 | 52 | 53 | 54 | 55 | 56 | 57 | 58 | 59 | 60 |
| | 18 | 61 | 62 | 63 | 64 | 65 | 66 | 67 | 68 | 69 | 70 | 71 | 72 |
| | 19 | 73 | 74 | 75 | 76 | 77 | 78 | 79 | 80 | 81 | 82 | 83 | 84 |
| | 20 | 85 | 86 | 87 | 88 | 89 | 90 | 91 | 92 | 93 | 94 | 95 | 96 |
| | | | | | | | | | | | | | |
| | 21 | 97 | 98 | 99 | 100 | 101 | 102 | 103 | 104 | 105 | 106 | 107 | 108 |
| | 22 | 109 | 110 | 111 | 112 | 113 | 114 | 115 | 116 | 117 | 118 | 119 | 120 |
| | 23 | 121 | 122 | 123 | 124 | 125 | 126 | 127 | 128 | 129 | 130 | 131 | 132 |
| | 24 | 133 | 134 | 135 | 136 | 137 | 138 | 139 | 140 | 141 | 142 | 143 | 144 |
| | 25 | 145 | 146 | 147 | 148 | 149 | 150 | 151 | 152 | 153 | 154 | 155 | 156 |

Supplement: Supplementary file 1 — Additional file 1: Figure S1. Representative IFNγ Elispot restimulation results and peptide pool matrix analysis. HLA-C*07 mice were immunized with full length MUC1 coding sequences and assay was performed on splenocytes. (a) 15mer. CD8-enriched cells (over 44% CD8+ cells) pooled from four immunized mice (HLA-C*07) were restimulated with pool of 15mers 1–24. The Elsipot is shown above, the matrix of 15mers 1–137 from the 24 pools covering the whole MUC1 protein is shown below. Pools above background in the Elispot (framed) are highlighted in the matrix. (b) 11mer. CD8-enriched cells (over 65% CD8+ cells) pooled from 7 immunized mice (HLA-C*07) were restimulated with pool of 11mers 1–25. The Elispot is shown above, the matrix of 11mers 1–156 from the 25 pools covering the whole MUC1 protein is shown below. Pools above background in the Elispot (framed) are highlighted in the matrix. [file 12967_2017_1254_MOESM1_ESM.pptx]

## Slide 1
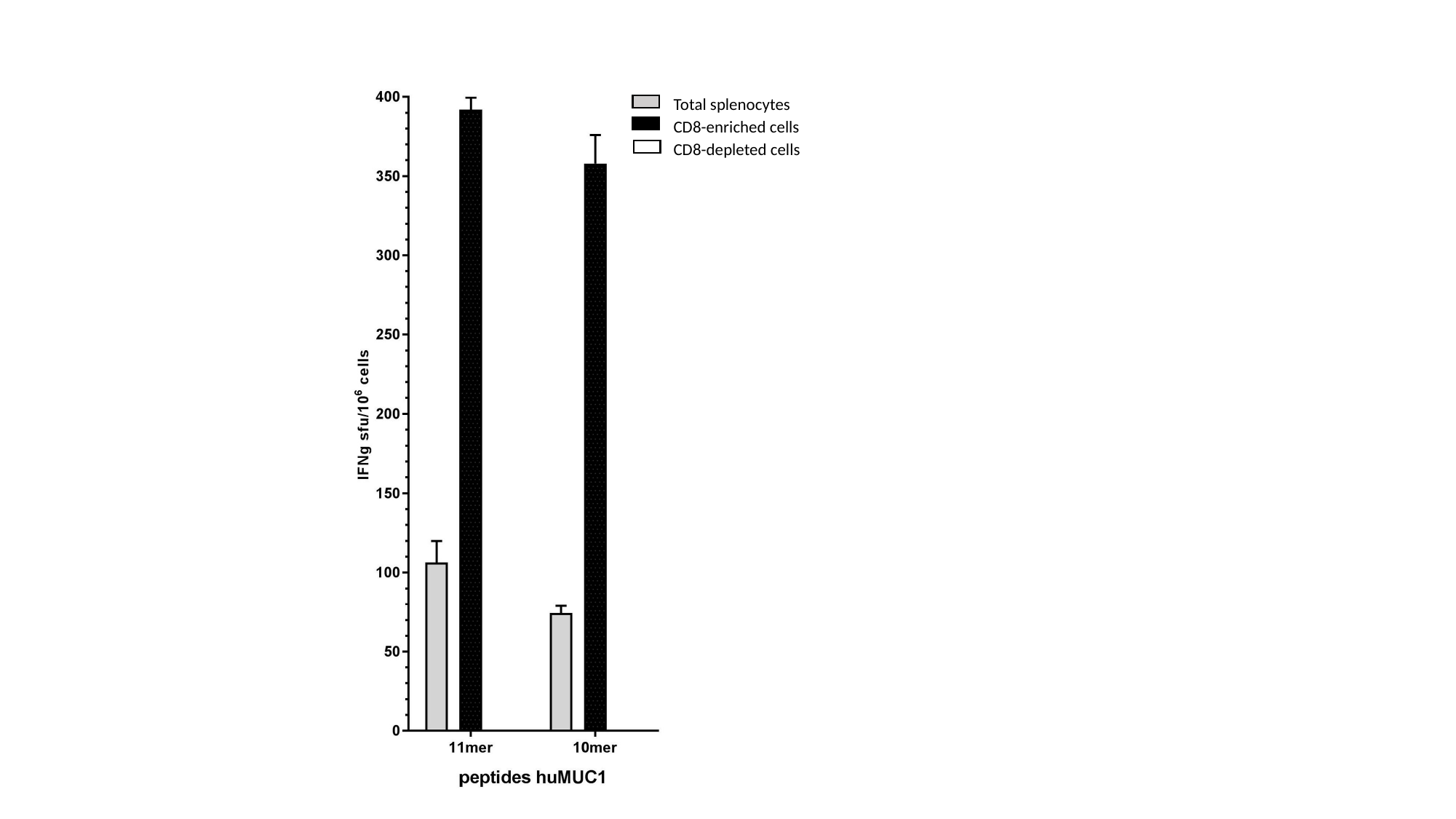

Total splenocytes
CD8-enriched cells
CD8-depleted cells

Supplement: Supplementary file 2 — Additional file 2: Figure S2. CD8-specific IFNγ response. Total splenocytes (grey with 23% of CD8+ and 14.4% CD4+), CD8-enriched cells (black with 84% CD8+, 0.2% CD4+) and CD8-depleted cells (white with 1.6% CD8+ and 19.6% CD4+) pooled from four immunized mice (HLA-B*35) were restimulated with 10mer FPARDTYHPM and 11mer IFPARDTYHPM. Only cells containing CD8+ cell showed an IFNg response (grey and black), the CD8-depleted cells (white, not visible) showed no IFNγ response. [file 12967_2017_1254_MOESM2_ESM.pptx]
