# Supplementary material for: Identification of new MUC1 epitopes using HLA-transgenic animals: implication for immunomonitoring
Source: J Transl Med. 2017 Jul 5;15:154. doi: 10.1186/s12967-017-1254-0 (PMC5499006; doi:10.1186/s12967-017-1254-0)
Supplement: Supplementary file 3 — Additional file 3: Table S1. Prediction of antigenic peptide by algorithms. The referenced MUC1 sequence was submitted to five algorithms for antigenic peptide prediction. Default settings were used for each of them. Numbers represent the rank for each indicated peptide. “-” indicates that the algorithm could not predict the peptide. Colored box indicate that the algorithm lacked one or more conditions for analysis (peptide length and/or allele). [file 12967_2017_1254_MOESM3_ESM.pptx]

## Slide 1
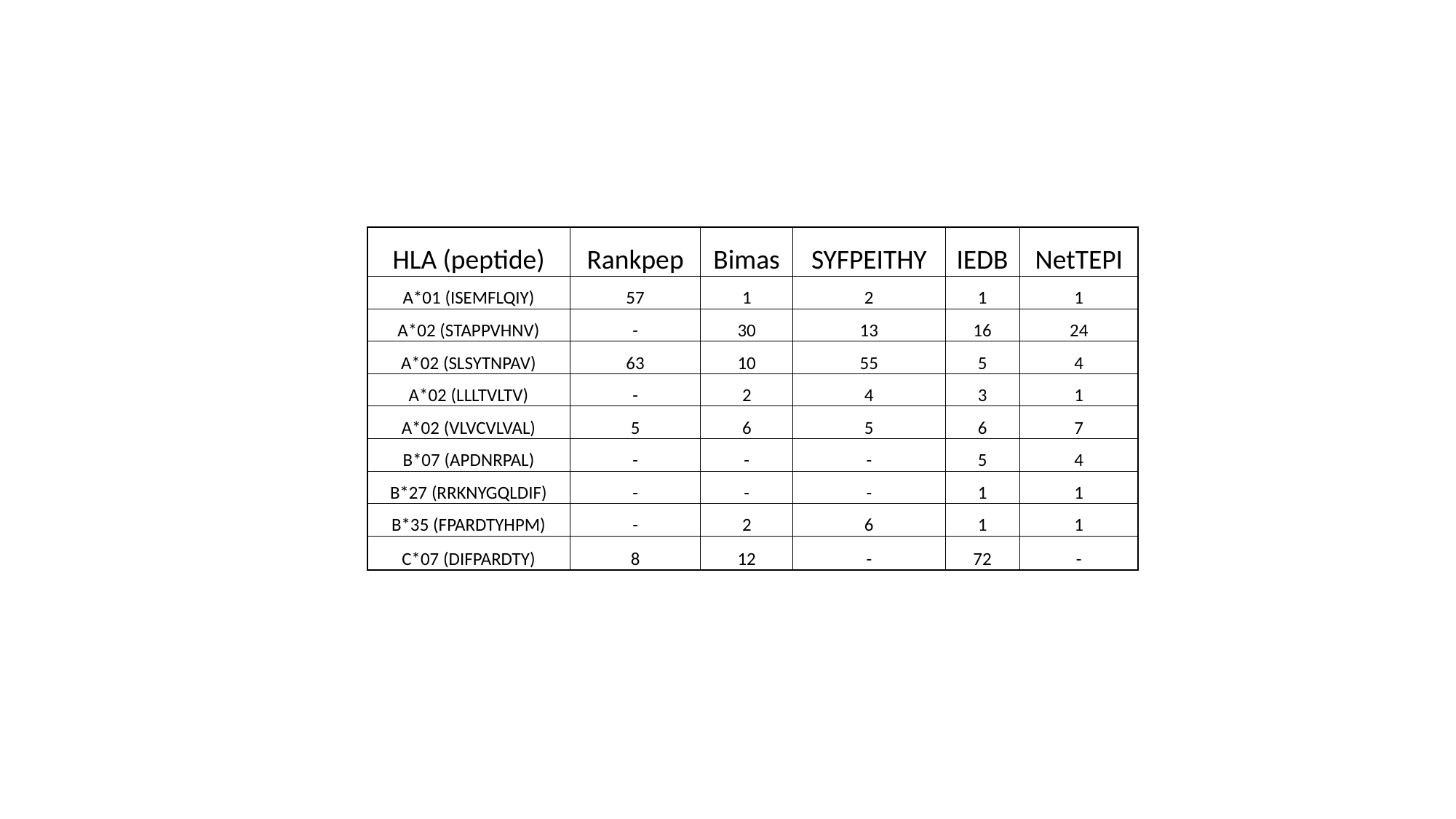

| HLA (peptide) | Rankpep | Bimas | SYFPEITHY | IEDB | NetTEPI |
| --- | --- | --- | --- | --- | --- |
| A\*01 (ISEMFLQIY) | 57 | 1 | 2 | 1 | 1 |
| A\*02 (STAPPVHNV) | - | 30 | 13 | 16 | 24 |
| A\*02 (SLSYTNPAV) | 63 | 10 | 55 | 5 | 4 |
| A\*02 (LLLTVLTV) | - | 2 | 4 | 3 | 1 |
| A\*02 (VLVCVLVAL) | 5 | 6 | 5 | 6 | 7 |
| B\*07 (APDNRPAL) | - | - | - | 5 | 4 |
| B\*27 (RRKNYGQLDIF) | - | - | - | 1 | 1 |
| B\*35 (FPARDTYHPM) | - | 2 | 6 | 1 | 1 |
| C\*07 (DIFPARDTY) | 8 | 12 | - | 72 | - |
